# Magnetoimpedance Biosensors and Real-Time Healthcare Monitors: Progress, Opportunities, and Challenges

**DOI:** 10.3390/bios12070517

**Published:** 2022-07-12

**Authors:** Valery Ortiz Jimenez, Kee Young Hwang, Dang Nguyen, Yasif Rahman, Claire Albrecht, Baylee Senator, Ongard Thiabgoh, Jagannath Devkota, Vinh Duc An Bui, Dao Son Lam, Tatiana Eggers, Manh-Huong Phan

**Affiliations:** 1 Laboratory for Advanced Materials and Sensors, Department of Physics, University of South Florida, Tampa, FL 33620, USA; valeryortizj@usf.edu (V.O.J.); keeyoung@usf.edu (K.Y.H.); dang3@usf.edu (D.N.); yasif@usf.edu (Y.R.); clairealbrecht9@gmail.com (C.A.); senator.bn.2018@wvwc.edu (B.S.); jagannath.devkota@netl.doe.gov (J.D.); daosonlamln@gmail.com (D.S.L.); 2 Department of Biomedical Engineering, University of South Florida, Tampa, FL 33620, USA; 3 Department of Physics, Faculty of Science, Ubon Ratchathani University, Warinchamrap, Ubon Ratchathani 34190, Thailand; 4 National Energy Technology Laboratory, Pittsburgh, PA 15236, USA; 5 Hue Central Hospital, Hue 52000, Vietnam; buiducanvinh@gmail.com; 6 Institute of Materials Science, Vietnam Academy of Science and Technology, 18 Hoang Quoc Viet, Ha Noi 10072, Vietnam

**Keywords:** magnetoimpedance, magnetic biosensors, healthcare monitors, COVID-19 detection

## Abstract

A small DC magnetic field can induce an enormous response in the impedance of a soft magnetic conductor in various forms of wire, ribbon, and thin film. Also known as the giant magnetoimpedance (GMI) effect, this phenomenon forms the basis for the development of high-performance magnetic biosensors with magnetic field sensitivity down to the picoTesla regime at room temperature. Over the past decade, some state-of-the-art prototypes have become available for trial tests due to continuous efforts to improve the sensitivity of GMI biosensors for the ultrasensitive detection of biological entities and biomagnetic field detection of human activities through the use of magnetic nanoparticles as biomarkers. In this review, we highlight recent advances in the development of GMI biosensors and review medical devices for applications in biomedical diagnostics and healthcare monitoring, including real-time monitoring of respiratory motion in COVID-19 patients at various stages. We also discuss exciting research opportunities and existing challenges that will stimulate further study into ultrasensitive magnetic biosensors and healthcare monitors based on the GMI effect.

## 1. Introduction

Since the turn of the 21st century, magnetic biosensor research has steadily grown year after year [1,2,3,4,5,6]. Magnetic phenomena such as the giant magneto-resistance effect, nuclear magnetic resonance, and superconducting quantum interference have often been proposed as the transducers of magnetic biosensors [2,7,8]. Although these magnetic phenomena offer valuable precision as transducers, their complicated measurement protocols, expensive equipment, and requisite for cryogenic temperatures have prevented them from being fully harnessed in the healthcare industry. This is where the giant magnetoimpedance effect, made prominent in 1994, found its footing in the world of biosensing and healthcare. High magnetic field sensitivity at room temperature coupled with classical and easy-to-model impedance–frequency–temperature relationships make the giant magnetoimpedance effect a qualified transducer of biometric data.

**Figure 1:**
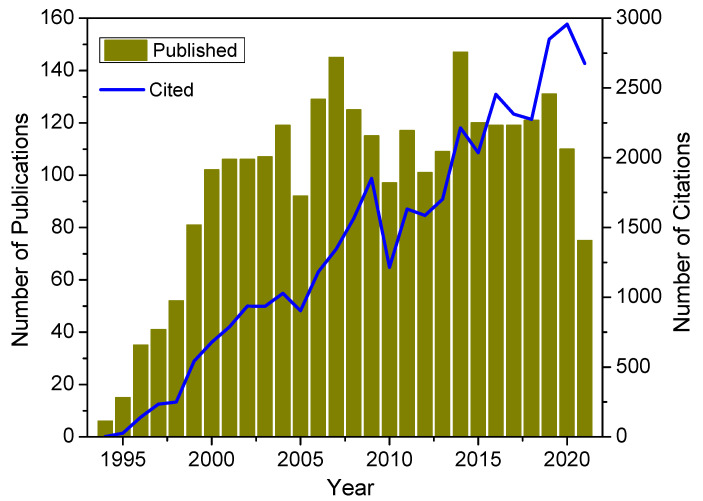
Number of published articles and citations per year in the field of magnetoimpedance materials and sensors. The data were collected from Web of Science with “magnetoimpedance” or “magneto-impedance” as a keyword.

The significant change in the impedance of a high (magnetic) permeability material when subjected to a small magnetic field was first reported in 1936 by Harrison et al. as an impedance magnetometer [9]. Much later, in 1991, Makhotkin et al. demonstrated a magnetic field sensor made of a soft ferromagnetic ribbon of FeCoSiB [10]. However, it was not until 1994, when two independent groups simultaneously published articles, that the phenomenological theory of the enormous impedance change of magnetic wires when experiencing a weak magnetic field was detailed [11,12]. At this point, the terminology “giant magnetoimpedance effect”, or GMI effect, was coined. Owing to its ultra-high magnetic field sensitivity, the GMI effect in soft ferromagnetic materials has been extensively explored and applied to both fundamental research and industrial applications. Excellent review articles on the GMI effect and its applications have been published thus far [13,14]. It is worth noticing in Figure 1 that the number of published articles per year greatly increased from 1994 to 2000 and slightly fluctuated between 2000 and 2020, while the number of citations has rapidly increased since 1994.

**Figure 2:**
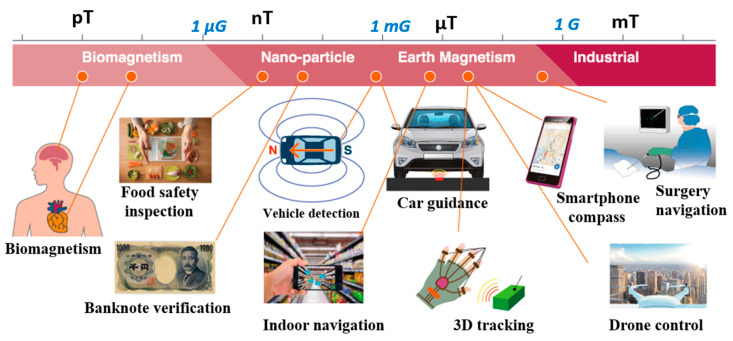
The potential applications of MI sensors proposed by Aichi Micro Intelligent Corporation. Reprinted with permission from Ref. [23]. Copyright 2022 Aichi Steel Corporation.

Magnetoimpedance (MI) biosensors are the products of a combination of the magnetoimpedance effect and electrochemical or affinity biosensors. Brought to the mainstream in 1994 by electrical engineers, the MI effect experienced a research peak in the fields of electrical engineering and materials science. Shortly after, the use of the MI effect for biological sensing applications was proposed by Mohri and coworkers [15]. Since then, condensed matter and materials science groups, electrical engineers, and biomedical researchers alike have conducted heavy research into using the GMI effect to detect biomagnetic particles and even record magnetic biometric data from the heart and brain [8,16,17,18,19,20,21,22]. Indeed, a wide range of ultrasensitive MI sensor prototypes and their potential applications, including biomagnetic sensing, have been proposed and developed by Aichi Micro Intelligent Corporation (see Figure 2) [23]. This technology is not without its shortcomings. As it stands today, GMI sensors suffer from two main limitations: (a) sensitivity and (b) quantification: two issues that researchers have attempted to solve both from a sensor design and a material engineering perspective. Sensor geometry, materials, structure, and operating frequency and current have been explored in order to optimize the performance of GMI-based sensors [18].

The implementation of GMI sensors in biomedical applications is largely achieved in conjunction with magnetic particles. Efforts to increase their sensitivity to the stray field emanating from magnetic particles is extensive and ongoing [24,25,26,27,28,29]. On the other hand, research into tailoring and functionalizing the properties of magnetic particles is equally important and complimentary to the development of GMI biosensors [30]. Magnetic particles can be functionalized to attach to specific molecules [24] or to carry drugs for targeted delivery in the human body [31], which requires a deep understanding of their magnetic properties and accurate detection to for applications in the field of nanomedicine.

In this review article, we focus on the short history of magnetoimpedance biosensors, how they have been improved over the past decade, and the state-of-the-art prototypes published within the past few years. We also highlight our recent developments in GMI-based medical devices for application in healthcare monitoring, including real-time monitoring of COVID-19 patients at various stages. Emerging opportunities and challenges in this rapidly expanding research field are also discussed to help guide future research and development of GMI-based biosensors for healthcare applications.

## 2. Basic Principles

The principle of MI-based biosensors is based on the detection of a small-magnitude (or “weak”) magnetic field by a change in impedance of a soft magnetic material. Essentially, an MI-based biosensor is a transducer that converts small changes of the magnetic field experienced by the sensing element into electrical signals. The sources of these small magnetic fields can be magnetic nanoparticles, red blood cells, magnetic signals from the brain, or the motion of tiny magnetic particles in media or tissue. MI biosensor applications range from qualitatively detecting the presence of small biomagnetic fields to providing a quantitative field measurement that can be translated into, for example, a particular concentration of magnetic particles. In this section, we first provide a concise review of the magnetoimpedance effect, as many great reviews on the subject have already been published [5,6,32,33,34]. Then, we outline the history of the earliest prototypes of magnetoimpedance-based biosensors sorted by their unique applications to biology and healthcare.

### 2.1. The Giant Magnetoimpedance Phenomenon

The GMI phenomenon refers to a large change in the complex impedance of a soft ferromagnetic conductor when subjected to an external static magnetic field. The change in impedance of a conductor consists of resistive (*R*) and reactive (*X*) components. The origin of the GMI effect can be demonstrated by the skin effect in classical electrodynamics and the circumferential magnetic permeability associated with circular domain wall movements. Generally, GMI materials possess large magnetic permeability and low resistivity, and understanding the enhancements and tradeoffs between these intrinsic material properties is crucial to optimizing the GMI effect for biosensor applications.

The complex impedance of a ferromagnetic conductor can be expressed as

(1) Z(μ,f,H)=R(μ,f,H)=+jX(μ,f,H)

where *R* is resistance, *X* is reactance, μ is magnetic permeability, f is operating frequency, H is the external magnetic field, and *j* is the imaginary unit. As Z(μ,f,H) and $\mu \left(f,H\right)=\mu^{\prime }-j\mu^{\prime \prime }$ both vary with *f* and *H*, the analysis of the GMI effect in a ferromagnetic conductor can be quite complex. Because of this, the GMI effect is typically categorized by frequency into three different regimes: low frequency, high frequency, and very high frequency. Many published articles have focused on the low-frequency response of the GMI effect; therefore, the scope of this section will mainly encompass the high-frequency response of soft magnetic materials.

The theory of magnetism and the dynamical response of magnetization can explain the origin of the high-frequency GMI effect. Beginning with Maxwell’s equations [35], we have:

(2) $$\nabla^{2}\vec{H}-\nabla \left(\nabla \cdot \vec{H}\right)=\mu_{0}\sigma \frac{\partial }{\partial t}\left(\vec{H}+\vec{M}\right)$$

where $\vec{H}$ and $\vec{M}$ are the external magnetic field and spontaneous magnetization vector, respectively. The dynamical response of the magnetization from an applied external magnetic field can be described by the Landau–Lifshitz–Gilbert (LLG) equation as [36,37,38]:

(3) $$\frac{\partial \vec{M}}{\partial t}=-\mu_{0}\gamma \left(\vec{M}\times \vec{H}\right)+\alpha \left(\vec{M}\times \frac{\partial \vec{M}}{\partial t}\right)\;$$

where γ is the gyromagnetic ratio, α is the damping parameter, and $\vec{H}$ and $\vec{M}$ are the external field and spontaneous magnetization within a domain, respectively. By applying the corresponding boundary conditions and solving the coupled Equations (2) and (3) [36,37,38,39,40,41], the longitudinal impedance of a cylindrical conductor for any frequency range can be expressed as:

(4) $$Z\left(\omega \right)=\frac{1}{2}kaR_{dc}\frac{J_{0}\left(ka\right)}{J_{1}\left(ka\right)}$$

where $k=\frac{\left(1-j\right)}{\delta }=\left(1-j\right)\sqrt{\mu f\mu_{\emptyset }\sigma }$, $\mu_{\emptyset }$ is the circular permeability, and $R_{dc}$ is the DC resistance of the magnetic wire.

Similarly, for a magnetic slab of thickness *2a*, the impedance can be expressed as:

(5) $$Z\left(\omega \right)=R_{dc}jka\;\coth(jka)\;$$

where $k=\frac{\left(1-j\right)}{\delta }=\left(1-j\right)\sqrt{\mu f\mu_{T}\sigma }$, $\mu_{T}$ is the transverse permeability, and $\;R_{dc}$ is the DC resistance of the magnetic slab.

The magnitude of the GMI effect of a magnetic microwire is defined by the change in *Z, R*, and/or *X* due to the external DC magnetic field. The figure-of-merit of GMI materials is the GMI ratio [14], and it is defined as follows:

(6) $$MI\%=\frac{Z\left(H\right)-Z\left(H_{max}\right)}{Z\left(H_{max}\right)}\times 100\;\%,\;$$

where *Z*(*H*) is the impedance at field *H*, and *H*_max_ represents the maximum value of the applied magnetic field. The magnetoresistance (MR) and magnetoreactance (MX) are defined in the same manner as in Equation (6), with *R* or *X* interchanged with *Z*. The magnetic field sensitivity (η) is defined as:

(7) $$\eta =\frac{d}{dH}\left(\frac{\Delta Z}{Z}\right)$$

The sensitivity of the magnetoresistance (MR) and magnetoreactance (MX) are defined in the same manner as Equation (7), with *R* or *X* interchanged with *Z*.

For the detection of magnetic particles, the sensitivity of the biosensor is defined as the difference between the maximum value in MI, MR, or MX (i.e., corresponding to the field value *H_k_*) of the test sample (*TS*) and reference sample (*ref*), which are calculated as:

(8) $$\Delta \eta_{\xi }={\left[\xi \right]}_{max,\;TS}-{\left[\xi \right]}_{max,\;ref\;}$$

where ${\left[\xi \right]}_{max}$ stands for $\xi =\frac{\Delta R}{R},\;\frac{\Delta X}{X},\;\frac{\Delta Z}{Z},\;$ which are the maximum values of the MR, MX, and MI ratios, respectively. These parameters are considered important figures-of-merit for assessing the sensitivity of an MI biosensor. In most studies,$\;\Delta \eta_{R}$, $\Delta \eta_{X}$, and $\Delta \eta_{Z}$ can also be denoted as MR, MX, and MI detection sensitivities, respectively.

### 2.2. Detection Principles

**Figure 3:**
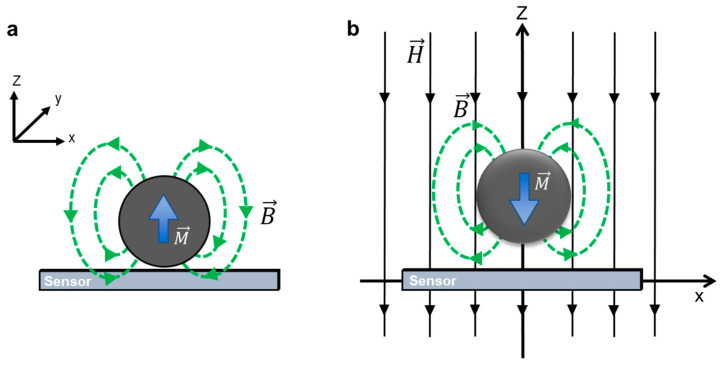
Schematics of stray magnetic field detection of magnetic beads (**a**) without an external magnetic field. Reprinted with permission from Ref. [43]. Copyright 2022 Elsevier, and (**b**) with an applied external magnetic field. Reprinted with permission from Ref. [27]. Copyright 2022 AIP Publishing.

The fundamental detection principle of an MI-based biosensor is the detection of the stray magnetic field of magnetic markers attached to the biomolecules of interest. To simplify this complex scheme, we can approximate the stray field of a magnetic biomarker as one generated by a single magnetized microsphere with a magnetic moment (*m*) symmetric about the center of the sphere (Figure 3). Then, the magnetic induction can be expressed as [42,43,44]:

(9) $$\vec{B}\left(\vec{r}\right)=\mu_{0}\vec{H}+\frac{\mu_{0}}{4\pi }\cdot \frac{3\vec{r}\left(\vec{r}\cdot \vec{m}\right)-\left(\vec{r}\cdot \vec{r}\right)\vec{m}}{r^{5}}$$

where $\vec{H}$ is the applied external magnetic field, $\;\mu_{0}$ is the magnetic permeability of free space, and *r* is the radial vector in spherical coordinates. Figure 3a,b show the schematics of stray magnetic field detection of magnetic beads without an external magnetic field [43] and with an applied external magnetic field [27], respectively.

From Equation (9), the in-plane magnetic field (${\vec{B}}_{x}$) at distance (*d*) along the *x*-axis from the center of the magnetic bead radius (*a*) and a small distance over sensing element (*t*) can be expressed as [27]:

(10) $$B_{x}=\mu_{0}M\frac{a^{3}\left(a+t\right)d}{{\left[{\left(a+t\right)}^{2}+d^{2}\right]}^{5/2}}$$

Similarly, the transverse magnetic field (${\vec{B}}_{z}$) can be expressed as:

(11) $$B_{z}=\mu_{0}M\frac{\left(2z^{2}-d^{2}\right)}{{\left[z^{2}+d^{2}\right]}^{5/2}}$$

where *M* = *m*/*V* is the magnetization of the magnetic bead.

**Figure 4:**
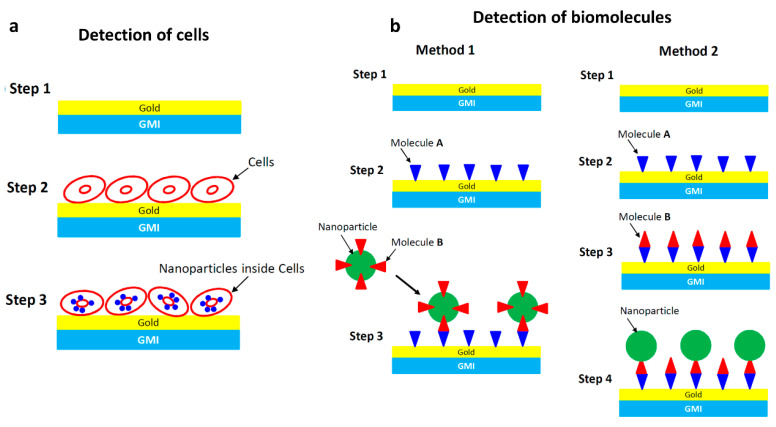
(**a**) Schematic illustration of the principle of detecting magnetic nanoparticles as magnetic labels inside cells; (**b**) schematic illustration of the principles of targeting and recognizing biomolecules. In Method 1, the magnetic nanoparticles are functionalized with target molecules (Molecule B), while in Method 2 the target molecules (Molecule B) are bound to the surface of the sensor.

The detection of stray magnetic fields emanating from magnetic beads or nanoparticles is the principal method behind potential applications of magnetoimpedance biosensing and medical diagnostics [16,29,44,45,46,47,48,49]. There are two main approaches to the measurement or detection of the magnetic particles: (i) detection directly on the surface of the sensor (Figure 4a) or (ii) detection from some distance away from the sensor (Figure 4b). For instance, a GMI sensor can be used as a probe of the presence of magnetic nanoparticles inside cells, which is the so-called magnetic label detection method (Figure 4a). A very thin layer of gold (~2 nm) is often coated on the surface of the sensing element to assure signal stability and biocompatibility [31].

For the detection of biomolecules using the GMI sensor and magnetic nanoparticles, two methods are often considered (Figure 4b). In Method 1, GMI measurements are first conducted on the sensitive element. Probe molecules (Molecule A) are then bound to the surface of the sensitive element, and GMI measurements are repeated. Magnetic nanoparticles, which are already functionalized with target molecules (Molecule B), will be bound to the probe molecules (Molecule A). In Method 2, target molecules (Molecule B) are first bound to the probe molecules (Molecule A), and the solution of nanoparticles then flows along the surface of the sensitive element. In both cases, the small magnetic fields generated by the nanoparticles that reach the surface of the sensitive element are detected using the GMI sensor [17].

Another new perspective is the contactless measurement of magnetic particles/biomagnetic samples. The stray magnetic fields of the magnetic beads and biomagnetic fields have recently been explored via ultra-sensitive MI-based sensors thanks to the promise of biosensing applications. Fodil et al. [29] designed an experimental setup to detect MNPs flowing through a microchannel at a distance, which is not coplanar with respect to the sensor, resulting in signal improvement. Additionally, the biomagnetic field of ileal musculature was measured ~1 mm below the sample using a gradio-magneto sensor. This detection approach can be developed for biomedical and medical diagnosis for in vivo samples [44]. Development and applications of these detection methods will be discussed in the following sections.

## 3. Early Sensor Prototypes (2000–2016)

### 3.1. Early Planar Prototypes

Amorphous ferromagnetic ribbons were first identified as potential weak magnetic field detectors in 1991 [10]. The earliest works on utilizing these magnetically soft ribbons as biosensors via the GMI effect were published a decade later by Kurlyandskya and coworkers [45,46,47]. In these works, rapidly quenched amorphous ribbons served as platforms for the detection of magnetic particles. Indeed, when a commercial ferrofluid or a suspension of Dynabeads was placed in close contact with the ribbons, their magnetoimpedance response increased due to their stray field interacting with the sensing element, and the field distribution of the magnetoimpedance widened.

A few years later, Yang and coworkers [48] demonstrated a Metglas ribbon as the sensing element of an MI-based biosensor for the detection and genotyping of human papilloma virus (HPV). This amorphous Metglas ribbon served as the sensing element, and it was micropatterned in a meander shape to increase the surface area for detection as well as for magnetic field sensitivity. The detection principle of this MI biosensor is to detect the stray magnetic fields of magnetic nanoclusters that label or tag HPV. The tagged HPV is then captured by specific probes on the surface of a microchannel in corresponding detection regions. When compared to the fluorescence method of HPV genotyping, the MI-based method had fewer steps, and the total assay time was significantly shortened.

The continuous flow detection of magnetic particles using an FeCoCrSiB ribbon and [FeNi/Ti]_3_/Cu/[FeNi/Ti]_3_ multilayer film was comparatively demonstrated in [49]. In this work, a 10 µL microfluidic chip was placed on top of the two sensing elements, and two different particle suspensions (Chemicell beads and Dynabeads) were pumped through the chamber as the magnetoimpedance was measured. While both the ribbon and thin-film sensor prototypes demonstrated a clear change in impedance when the particles entered the chamber, the results were not easy to reproduce. Although the use of a transmission line to measure the impedance improved the noise level in the experiments compared to prior work, the authors acknowledged the need for signal filtering to bring the sensitivity and reproducibility of these biosensor prototypes up to commercial standards.

**Figure 5:**
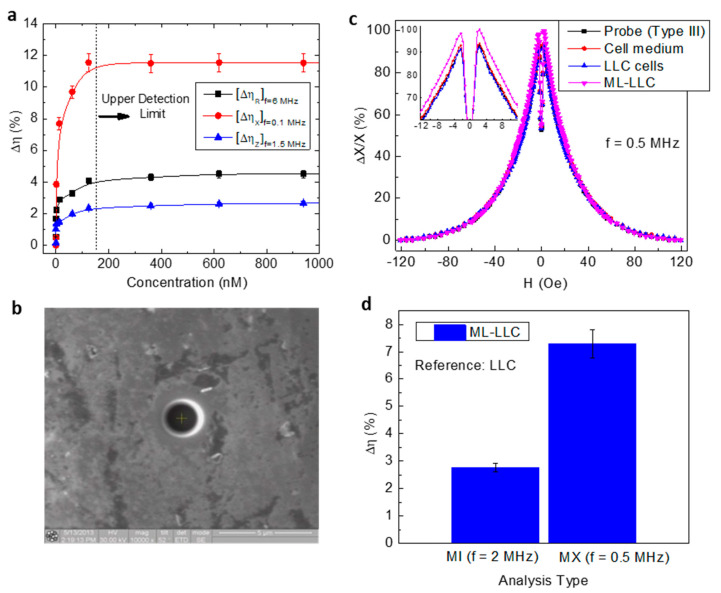
(**a**) Fe_3_O_4_ particle concentration dependence on MR, MX, and MI detection sensitivities and (**b**) SEM image of a hole on the ribbon. (**c**) Magnetic field dependence on MX ratio for a hole-based MX biosensor with cell medium, LLC cells, and magnetically labelled LLC cells (ML-LLC). (**d**) MI- and MX-based detection sensitivities of the probe for ML-LLC with reference to LLC.

While most efforts have been focused on developing a biosensor based on the MI effect, which has limited sensitivity, Devkota et al. [50,51,52] showed that by exploiting the MR and MX effects, it is possible to improve the sensitivity of the biosensor ($\Delta \eta_{R},\Delta \eta_{X}$) by up to 50% and 100%, respectively. The increase of $\Delta \eta_{R}$, $\Delta \eta_{X}$, and $\Delta \eta_{Z}$ with increasing concentration of iron oxide nanoparticles (Figure 5a) can be attributed to the increase of transverse susceptibility $\mu_{T}$ due to the strong coupling of the magnetic fringe fields of the nanoparticles to the AC transverse magnetic field. This coupling becomes independent of iron oxide nanoparticles after the concentration of nanoparticles exceeds a critical amount, and no further increase in Δη is therefore obtained. The authors also demonstrate that patterning the ribbon surface with nano/micro-sized holes is an effective way to improve the detection sensitivity of a ribbon-based MI biosensor [53]. This is particularly important as improvement in detection sensitivity can lead to highly sensitive detection of bioanalytes tagged to magnetic markers or cells that have taken up magnetic markers. Indeed, the authors fabricated a novel sensor probe by patterning four holes, each of dimension 2 μm × 2 μm, on a soft ferromagnetic ribbon using a focused ion beam (FIB) [54]. They analyzed the MI and MX responses for the probe itself and with 10 μL of the cell medium (as the control), unlabeled Lewis lung carcinoma (LLC) cancer cells, and magnetically labelled Lewis lung carcinoma (ML-LLC) cells (Figure 5b–d). The results showed that the sensor probe, cell medium, and label-free LLC cells did not have significant difference in their MI profiles (MI and MX ratios), while the ML-LLC cells had higher values. This demonstrates the possibility of using a hole-based MI biosensor to separate ML-LLC cells from unlabeled LLC cells. A GMI sensing platform could thus be developed as the new generation of diagnosis systems for reliable and quick biodetection at room temperature that can also be used as a new, low-cost, fast, and easy pre-detection method prior to magnetic resonance imaging (MRI).

Early biosensor prototypes utilizing amorphous ferromagnetic ribbons certainly encouraged further study of MI-based biosensors. However, when considering the incorporation of amorphous ribbons into magnetic biosensors for applications in the healthcare industry, some problems arise. For one, due to the rapid quenching technique used to prepare them, the physical and magnetic properties of the ribbons can vary significantly between different sections of the same batch [55]. Thus, mass-produced ribbons used as biosensing elements in commercial products would require individual characterization and adjustment due to differences in sensitivity. Furthermore, as the operating frequency increases, the formation of eddy currents leads to a sharp increase in heat losses, which can deteriorate the magnetic properties of most commercial ribbons, for which the thickness is in the range of tens of microns [56,57].

While the planar geometry of amorphous magnetic ribbons is well-suited to magnetic particle detection, which is the most popular application of magnetoimpedance biosensors, the requirement of miniaturization forces sensing elements toward smaller and smaller sizes. This is where thin-film structures exhibiting the magnetoimpedance effect begin to outshine amorphous ribbons as sensing elements. While miniaturization itself leads to a new set of challenges, ribbons are essentially disordered bulk structures and cannot meet the standards of mass reproducibility desired for commercial production. On the other hand, thin-film growth and patterning techniques have long been established to produce consistent results. It is clear that thin-film-based magnetoimpedance sensors serve as a way to overcome some of the limitations of ribbon-based sensors, and are further explored in the remainder of this section.

Some of the earliest thin-film biosensor prototypes used the GMI effect of FeCuNbSiB/Cu/FeCuNbSiB thin-film trilayer systems [58]. The composition FeCuNbSiB is of the FINEMET family, which are well known as rapidly quenched ribbons. In thin-film form, FINEMET structures were deposited via RF sputtering and annealed to induce ferromagnetic Fe–Si nanocrystalline grains within a ferromagnetic matrix that remained amorphous. Indeed, it is this microstructure that makes the magnetoimpedance effect of FINEMET films highly sensitive to weak magnetic fields and thus well-suited to MI-based biosensors. The uniformly distributed nanograins embedded in the amorphous matrix assure near-zero magnetostrictive anisotropy. Moreover, the magnetocrystalline anisotropy in nanocrystalline materials is averaged out, which also contributes to its high sensitivity to weak magnetic fields [59].

In 2009, Volchkov and coworkers [60] introduced another MI biosensor prototype based on a NiFe trilayer structure. In the world of spintronics, NiFe, or permalloy, is a well-studied and often used soft magnetic material. In this work, the authors varied the width of the sensing element, which was either a single NiFe film, a rectangular NiFe/Cu/NiFe trilayer structure, or a NiFe/Cu/NiFe trilayer structure where the center Cu conductor was narrower than the NiFe layers. The sensors were operated at frequencies in the upper hundreds of MHz, where the MI effect was found to be the largest. The authors discovered a tradeoff between the width of the sensing element and the observed MI effect; that is, when increasing the width of the sensing element to increase the detection area, the MI effect decreased. This work illustrates the importance of geometry when designing magnetoimpedance biosensors while keeping in mind the size of the particles to be detected. Wang et al. later reported a multilayered NiFe/Cu/NiFe meander film for MI-based biosensing grown using micro-electro–mechanical systems (MEMS) [61,62]. The authors noted that these films’ maximum GMI ratio was observed at significantly lower frequencies than most ribbons, a desirable feature in biosensing applications. A quantitative study of the detection of Dynabeads Protein A was performed, demonstrating the accurate detection of Dynabeads down to a concentration of 0.1 μg mL^−1^ at a frequency of 1.4–1.5 MHz. These studies further highlight the importance of the geometry of the sensing element, but also demonstrate the potential of thin-film-based MI biosensors for accurate quantification of magnetic biomarkers

In summary, planar geometries of magnetoimpedance-based sensors are available in a wide variety of compositions and shapes, all of which have the advantage of a large surface area. A large sensing area is ideal for the detection of magnetic particles and biomarkers, especially in contact-based measurements. While amorphous ribbons possess high sensitivity, their production methods limit mass reproducible sensors without the need to individually characterize them. Thin-film-based biosensors offer a clear alternative to mass-reproducible sensors, and the growth techniques are compatible with miniaturization standards. Two major issues persist in early MI planar sensors: sensitivity and quantification. Sensitivity is limited by the composition and operating frequency of the sensing element, and due to the limited sensitivity, quantification is a challenge, especially in an unshielded environment. Both issues are addressed in more recent works, as we discuss in Section 4. In the following subsection, we focus on MI sensor prototypes based on soft magnetic wires and compare their performance and applications with that of planar MI sensors.

### 3.2. Early Wire-Based Prototypes

So far, much of this review has focused on MI biosensors for the detection of stray magnetic fields emanating from magnetic particles that could be tagged to biomolecules. However, there is another equally important application of MI biosensing, and that is the detection of small-magnitude magnetic fields produced by biochemical current flow or the presence of small quantities of ferromagnetic contaminants [63]. For example, the motion of red blood cells in the body, the nervous system, and the movement of neurons within the brain all produce magnetic fields. Indeed, prototypes of wire-based MI biosensors used to detect weak biological magnetic fields predate many of the planar particle-detection prototypes mentioned in the previous section, and, additionally, wire-based MI protypes have consistently shown greater field sensitivity than planar prototypes.

The scientists responsible for constructing the first MI sensor are L. Panina, K. Mohri, T. Uchiyama, and coworkers [64]. In their pioneering 1995 work, the authors fabricated a highly sensitive magnetic field sensor using a 200 MHz resonant multi-vibrator bridge circuit that combined two CoFeSiB amorphous microwires and two field-effect transistors. It is worth noting again that this wire geometry sensor prototype predates the ribbon- and film-based sensors mentioned in the previous sections, which also came with extensive and custom integrated circuit elements to reduce noise and improve quantitative measurements.

Shortly thereafter, Uchiyama and coworkers demonstrated the use of this MI biosensor to detect the position of brain tumors in rats after injection with a solution of 25-nm magnetite nanoparticles dispersed in agarose [65]. The authors found that their MI sensor was able to detect the position and size of the tumor and produce a basic topographical map of the tumor by detecting the stray field produced by the magnetite particles embedded in it.

**Figure 6:**
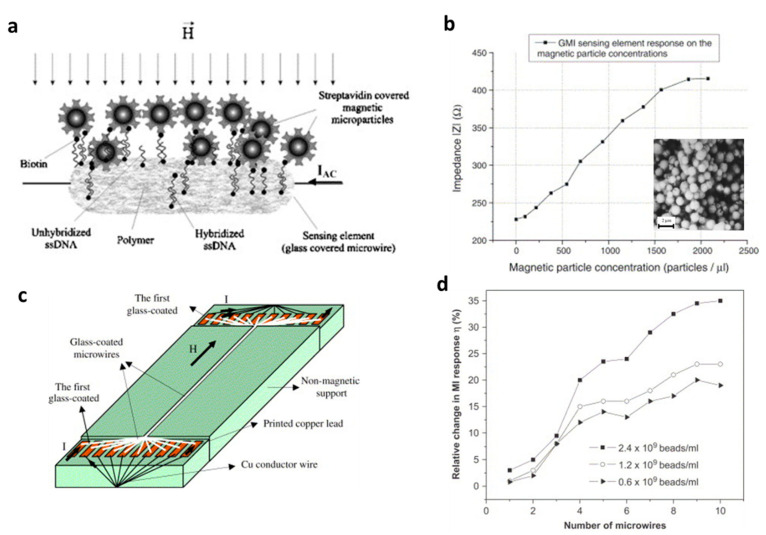
(**a**) The principle of a GMI-based magnetic biosensor using the ssDNA hybridization phenomenon as an example; (**b**) impedance response of the sensing element on the magnetic particles concentration. Reprinted with permission from Ref. [24]. Copyright 2022 Elsevier; (**c**) schematic design of the multiwire-based MI device; and (**d**) relative change in MI response as a function of the number of active glass-coated microwires for different bead concentrations. Reprinted with permission from Ref. [25]. Copyright 2022 Elsevier.

Chirac et al. [24] proposed an MI-based biosensor using a combination of ssDNA hybridization capture with streptavidin-covered magnetic microparticles (Figure 6a). The sensing element was a glass-covered CoFeSiB microwire, and its impedance was simply measured in a four-point configuration with a probe frequency around 10 MHz. Concentrations as low as 25–30 magnetic particles/µL could be detected in this arrangement (Figure 6b). Expanding on this work, Chirac and coworkers fabricated an MI biosensor prototype based on an array of glass-coated amorphous microwires and applied it to the detection of commercial polymer-based magnetic particles (Figure 6c,d) [25]. It was found that the number of active microwires enlarged the relative change in MI response, and this microwire arrangement could be used as an MI biosensor.

In the same year, Chiriac and coworkers published a systematic study on the detection of different sizes of magnetic particles (four ranges of sizes: 40–60 µm, 60–100 µm, 100–150 µm, and 150–300 µm) by a single CoFeSiB glass-coated microwire to predict whether these types of particles could function well as magnetic markers [26]. The results indicated that all sizes of magnetic particles in this study produced a notable and easily detectable MI response. The largest increase in MI effect, about 43%, was found with microparticles in the size range of 150–300 µm and a detection configuration of DC field parallel to the wire and measurement frequency of 10 MHz.

In 2013, Fodil et al. [29] combined microfluidics with an MI micro-magnetometer to detect 20 nm iron oxide nanoparticles, which could be functionalized for biomarkers. The micro-magnetometer was an MI biosensor based on a 40 µm CoFeSiBNb microwire sensing element at a measurement frequency of 15 MHz. In this experimental configuration, the microfluidic channel was a small distance away from the sensor. The authors showed two successive flows of MNPs measured by the MI microwire sensor, and the peak of the detected magnetic field was at a 180 nL volume of MNP flowing near the sensor at 4.3 mm/s. As a next step in the development of the sensor, Fodil and coworkers [66] reported the in-flow detection of a very low concentration of superparamagnetic nanoparticles (as small as 5.47 × 10^−9^ mol), which was also confirmed theoretically in a subsequent publication [67].

Wire-based sensors lack the surface-area planar geometries but make up for it in increased sensitivity for contactless measurements. Early prototypes not only detected the stray field of magnetic particles of different sizes but also detected biomagnetic fields, which ribbon and thin-film sensors cannot do. Wire-based sensor sensitivity can be further improved in combination with integrated circuit elements, novel sensing arrays, and other approaches that are discussed in the following section.

## 4. Current Magnetoimpedance Biosensors and Healthcare Monitors (2016–Now)

### 4.1. Detection of Magnetic Particles

The structure of a thin-film MI sensor significantly impacts its sensitivity. In general, two types of thin-film structures are being studied: straight line films and meander films. In both cases, different multilayer structures have been investigated with different levels of success that are summarized below.

Presently, some of the most widely studied materials in this field are permalloy-based films, largely due to their well-established deposition techniques that allow for consistency across different films [68]. In general, the thickness of the material must match the skin depth associated with the operational frequency to obtain a significant MI ratio. For permalloys probed at a frequency in the few to tens of MHz, the corresponding skin depth are in the micrometer range, which is large compared to the minimum thickness one can achieve with sputtering techniques without degrading its magnetic properties [68].

It has been well-established in MI research that a metallic conductor placed between the two ferromagnetic layers increases the MI effect of the ferromagnetic conductor [28,69,70,71,72,73,74,75]. Therefore, multilayer structures are a research direction that achieves the desirable thickness for large skin-depth variations to enhance the MI effect of thin-films. A popular structure that incorporates these findings is FeNi/Cu/FeNi sandwich films, where Cu separates FeNi multilayers and serves as the central conductive spacer [76]. Including an insulator between the metallic and ferromagnetic layers has also been shown to further increase this effect as a consequence of changing the distribution of the electromagnetic field in the film to promote a change in impedance upon the application of an external field [77]. Kurlyandskaya et al. reported that the closer the conductivity of the spacer to that of the film, the more evenly distributed the electromagnetic field will be, which is conducive to a larger MI effect in the material [69]. The reported MI ratios for these structures vary with composition, thickness, length, and shape; these results are summarized in Table 1.

**Figure 7:**
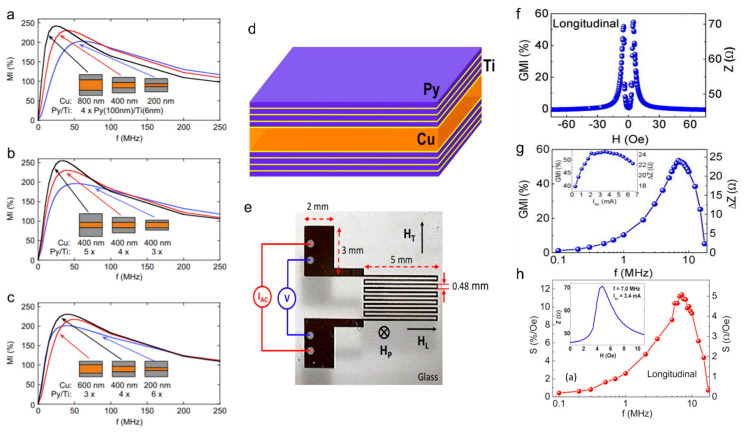
Compilation of results showing optimization of a multilayer structure’s MI ratio as a function of frequency. (**a**–**c**) The optimization of the dimensions of a single strip [Py/Ti]/Cu/[Py/Ti] film (i.e., sensing element) in a meander structure. Reprinted with permission from Ref. [68]. Copyright 2022 Elsevier. (**d**) The film was grown in a layered structure [Py/Ti]/Cu/[Py/Ti]. Reprinted with permission from Ref. [71]. Copyright 2022 AIP Publishing. (**e**) The meander has 12 segments, each one 0.16 mm wide and 5 mm long, and the separation between segments is 0.48 mm. GMI ratio as a function of magnetic field (**f**), frequency (**g**), and ac current (inset of (**g**)). (**h**) Field sensitivity of GMI as a function of frequency, with an inset showing *Z*(*H*).

There are several factors that must be carefully considered when choosing the composition of such structures. Current research has shown that the choice of nonmagnetic conductor and its thickness impacts the MI response of the films [68]. The thickness and dimensions of the ferromagnetic layers also plays an important role that will set a limit to the sensitivity of the film. Figure 7a–c shows the dependence of the MI ratio on the dimensions of the different layers. This shows that the size of the central Cu layer relative to the size of the magnetic multilayers impacts the MI ratio; therefore, it should be a property that is carefully controlled to optimize the sensitivity of the film. In summary, the choice of structure should be prudently considered based on the desired application, as it will have a considerable impact on sensitivity.

Another widely studied structure for thin-film MI sensors is meander type films. While the structures of meanders are more complex, they offer an advantage over single-line strip films due to the formation of a transverse AC magnetic field on each segment of the meander [75,77] that may result in an electromagnetic coupling effect that enhances the MI effect. Meanders incorporate the same multilayer structures used in straight-line MI sensors but in a compact form ideal for applications while still maintaining a significant MI ratio (Figure 7d,e). Additionally, the AC magnetic field may prove useful in magnetizing any magnetic particles near the film. This is particularly important when working with superparamagnetic nanoparticles, where the magnetization process may allow for the detection of these particles without the necessity of an external magnetizing field [77]. An additional parameter that may also be studied is the operating current of the sensors, which has been shown to impact sensor sensitivity (Figure 7f–h).

The main application of MI-based biosensors has been for the detection of biomarkers, such as magnetic particles. Several works have been dedicated to the detection of small quantities of superparamagnetic beads [77,78,79] due to their potential as biomarkers that make it possible to employ MI sensing elements as biosensors. It has been shown that thin-film-based biosensors have been used to detect as few as 10 magnetic beads [77]. Wang et al. [77] demonstrated how a meander-shaped MI sensor can be used to detect alpha-fetoprotein (AFP) by conjugating superparamagnetic Dynabeads. They used a Cr/Cu/NiFe/Cu/NiFe/Al_2_O_3_/Cr/Au meander structure. The Al_2_O_3_ layer served as an insulation layer between the sensing element and the immunoplatform (Au). The authors showed that the sensor is capable of detecting concentrations of AFPs as small as 1 ng/mL although based on their previous work detecting Dynabeads [61], they reported that their detection limit was closer to 1 pg/mL.

**Figure 8:**
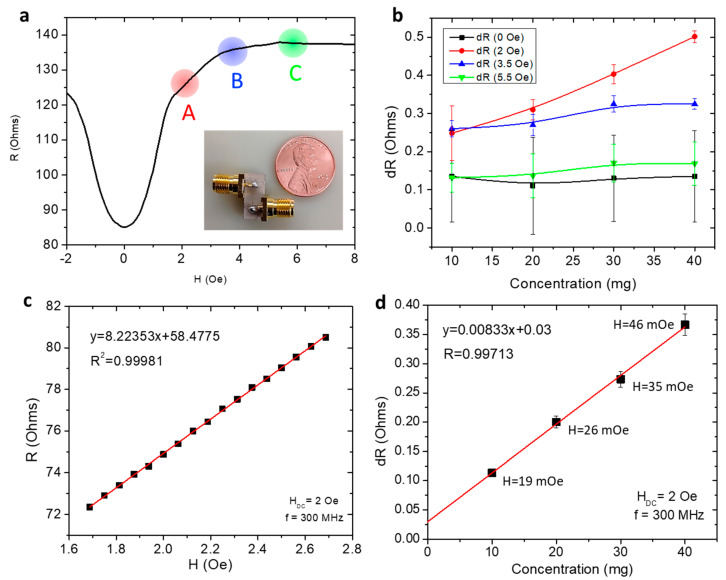
(**a**) The low-field (-4 Oe to 9 Oe) GMI curve demonstrating the regions measured during nanoparticle detection. ‘A’ corresponds to *H_DC_* = 2 Oe; ‘B’ is *H_DC_* = 3.5 Oe (~*H_K_*: the anisotropy field); and ‘C’ is *H_DC_* = 5.5 Oe (>*H_K_*); (**b**) the change in resistance (dR) in a soft ferromagnetic Co-rich wire when exposed to different quantities of nanoparticles relative to the resistance with no nanoparticles present. Measurement was performed at each of the DC fields *H_DC_* = 0 Oe, 2 Oe, 3.5 Oe, and 5.5 Oe; (**c**) linear fit of the low-field region of the MI curve allowed us to calculate the stray field of Fe_3_O_4_ nanoparticles; and (**d**) the linear change in resistance (d*R*) for *H_DC_* = 2 Oe indicates that this MI-based microwire sensor can be used to detect small concentrations of magnetic nanoparticles.

While most of the studies attempted to demonstrate the capacity of GMI biosensors in detecting weak magnetic signals from low-concentration magnetic nanoparticles that are used as magnetic biomarkers, these biosensors are unable to provide quantitative values of the stray fields created by the magnetic nanoparticles. To address this, Phan’s group developed an effective method based on the linear-field GMI response, which can detect and determine stray field values of magnetic (Fe_3_O_4_) nanoparticles at different concentrations (Figure 8). A single Co-rich microwire was used as a sensing element (inset of Figure 8a). To optimize the GMI biosensor’s performance, they applied an external magnetic field (~2 Oe) below the anisotropy field (*H_K_*) of the microwire, which brought the impedance change to a linear detection regime (Figure 8a). By fitting the linear region of the GMI curve (here only the *R* vs. *H* dependence was considered; Figure 8b,c), they were able to determine the minimum detection capability to be about 19 mOe from 10 mg of Fe_3_O_4_ nanoparticles placed 1 mm from one end of the microwire (Figure 8d). Future studies should focus on increasing the sensitivity of the microwire further to precisely detect even lower fields. This can be achieved in a myriad of ways, including optimizing the composition of the wires or modifying the domain structures with stress, heat treatment, and magnetic field annealing. Further improvements to signal processing can be achieved by carefully engineering circuits [80,81], understanding noise behavior [82], and exploring more complex sensor designs that integrate multiple sensing elements for signal filtering [20,21,22,46,83,84,85,86,87].

### 4.2. Biomagnetic Field Detection

Biomagnetic fields are magnetic fields produced from living systems, and they have been a hot topic of interest due to their small but significant effects, such as their direction-seeking effect on bird migration, their effect on the movement of bacteria, and as important signal sources from the human heart and brain. Therefore, biomagnetic measurement of magnetic fields is a crucial tool in investigating the functional organization of some human organs. Due to their extremely high field sensitivity down to the picoTesla level at room temperature, GMI-based sensors have been employed for biomagnetic field detection. The localized biomagnetic fields generated by smooth muscle cells, cardiogram signals, and smooth muscle tissue samples taken from a guinea pig have been detected using an MI sensor without a magnetic shield [19,20,46]. The biomagnetic signal from ileum musculature samples have been measured to be up to several nT [46]. Recently, numerous studies have suggested that the superior field sensitivity of GMI-based sensors is highly promising for magnetocardiography (MCG) and magnetoencephalography (MEG) [22,32].

**Figure 9:**
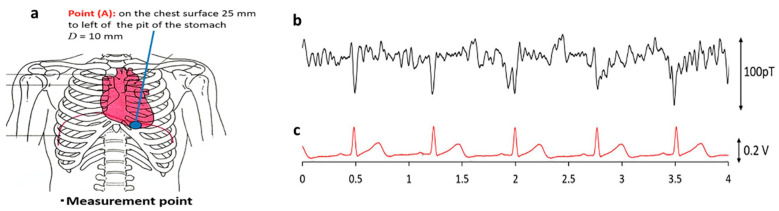
(**a**) Measurement location of MCG, which was 25 mm to the left of the pit of the stomach and 10 mm from the surface. (**b,c**) Real-time recordings of MCG and ECG signals measured by a peak-to-peak VD-type MI gradiometer without any magnetic shielding equipment. Reprinted with permission Ref. [86]. Copyright 2022 Elsevier.

Measurement of an MCG signal using an off-diagonal MI gradiometer in an unshielded environment at room temperature was reported in 2017 [83]. The sensor head of the gradio-magneto sensor was located 10 mm from the body surface of the test subject. Simultaneous measurements of electrocardiogram (ECG) and MCG signals were performed. Because of the chest movement from the test subject, the shown signal was averaged over more than 50 cycles. An active magnetic shielding system was developed for this gradiometer, which effectively reduced environmental magnetic noise around the sensor head and reduced the number of averaging cycles from 50 to 25 [84]. Moreover, Mohri et al. [22] utilized amorphous wire MI sensors integrated with CMOS to measure the back MCG from the left scapula of a test subject. The obtained signals showed excellent results when compared to simultaneous measurements of ECG and MCG. Ma and Uchiyama [85] later developed a new type of MI gradiometer consisting of a pair of CoFeSiB amorphous wires and a pick-up coil, which used a peak-to-peak voltage detector by synchronized switching. With this highly sensitive sensor, they performed MCG and MEG at room temperature (Figure 9) with an average of 10–15 cycles in an unshielded environment and achieved a noise amplitude of 100 pT [86].

Real-time brain activity measurement was carried out using a highly sensitive MI sensor without any magnetic shielding at room temperature [21,87,88]. Uchiyama and coworkers set up a GMI-based gradiometer to measure a small magnetic field 5 mm from the back left of the subject’s head. The compared signals were ~500 pT in magnitude between eye opening and eye closing with respect to the background field. An analogous experiment was reported in [87], where an auditory evoked field (AEF) brainwave was probed using a highly sensitive MI sensor (picoTesla resolution) in a normal environment. The authors showed a difference in the received signals when the test subject opened or closed his/her eyes [87]. This real-time monitoring of brain activity signals supports the findings of the gradiometer measurement. Recent studies have explored alpha rhythm and visual event-related field (ERF) measurements using a high-performance MI sensor system in an unshielded environment [88]. The authors showed the difference in the measured MEG and EEG signals simultaneously when the test subject opened or closed his/her eyes. This noninvasive real-time monitoring of human biomagnetic fields using a highly sensitive MI-based sensor could be applied for brain activity measurement.

### 4.3. Microfluidics

**Figure 10:**
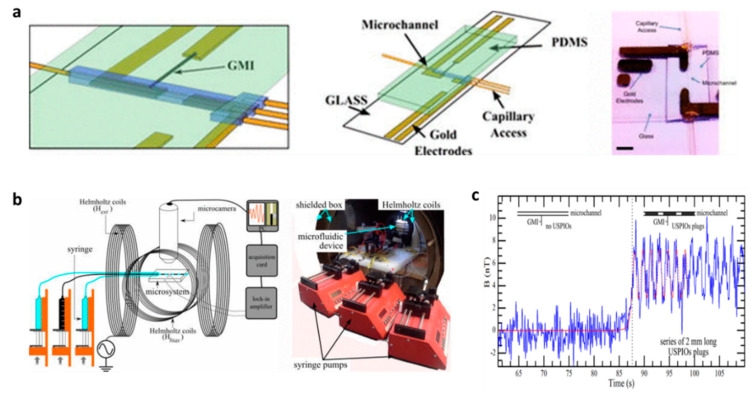
(**a**) Left to right: 3D schematics of the microfluidic system showing the PDMS micro-channel on one side of the glass substrate, the GMI microwire with electrical connections on the other side, and a picture of the actual device. (**b**) Picture of the experimental bench where one can see the programmable syringe pumps, the shielded box, the Helmholtz coils, and the microfluidic system. (**c**) Measured magnetic signal before and after (black dotted, *t* = 87.5 s) injection of the USPIOs plugs with 5.47 × 10^−9^ mol contents (2 mm long-20 nL volume with a molar concentration of 230 mmol/L). Reprinted with permission from Ref. [67]. Copyright 2022 AIP Publishing.

The superior magnetic field sensitivity of MI biosensors makes them attractive sensors in sensitive microfluidic platforms. Recently, the detection of biofunctionalized magnetic particles using a variety of MI geometries as sensing elements has been explored from the viewpoint of biomedical and clinical diagnostics. In 2016, the in-flow detection of ultra-small magnetic particles (20 nm) with a nanomolar concentration of superparamagnetic nanoparticles was achieved using a Co-rich microwire-based sensor (see Figure 10). The experimental results were theoretically validated with a model of the magnetization of a linear homogeneous isotropic material [67]. Later, an off-diagonal GMI biosensor integrated with a simple microfluidics chip was employed to detect antibody and alpha-protein (AFP) antigens labelled with magnetic beads in concentrations as low as 100 fg/ml under an external magnetic field. The experiment reveals that linear correlation with the AFP concentration is a useful approach for detecting cancer biomarkers [89].

Feng et al. employed a GMI-based immunoreaction platform to detect the stray magnetic field and variation of magnetic signal from immunomagnetic beads [90]. In this configuration, a meander-line structured Co-based ribbon was integrated onto a microfluidic chip fabricated by MEMS. The integrated microfluidic chip consisted of incoming inlets and incubation and reactive chambers used as the biomarker detection system. In this detection scheme, prostate specific antigen (PSA) was detected at a concentration of 0.1 ng/mL, which is quite promising for further quantitative analysis. Recently, Melnikov et al. fabricated FeNiCu multilayer nanostructures that were used for the detection of magnetic particles in a blood-vessel-like structure [28]. The measurement of stray fields generated by iron-oxide microparticles under an external magnetic field was carried out using the longitudinal MI effect. The position of magnetic composite samples mimicking a thrombus (blood clot) was performed experimentally and modelled theoretically using COMSOL. This experiment is promising for thrombosis evaluation and therapy [28].

### 4.4. Real-Time Healthcare Monitoring of Patients with Respiratory Illness or COVID-19

**Figure 11:**
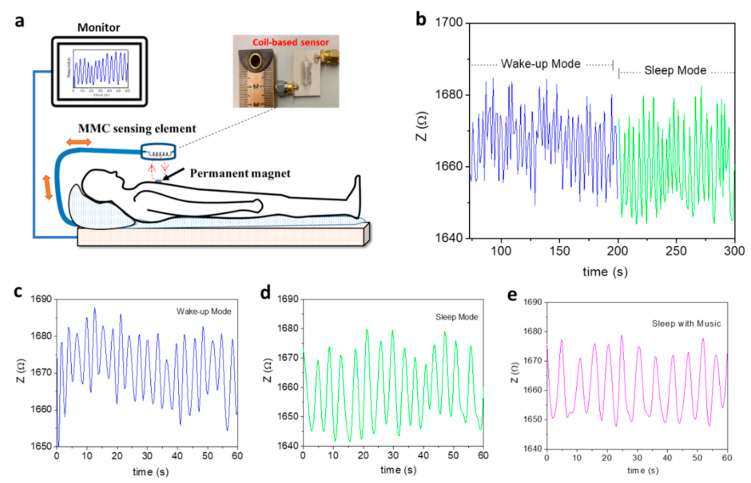
(**a**) Schematic for the respiratory motion test and results; inset is an image of the soft-magnetic-coil-based sensor probe; (**b**) breathing patterns of a 42-year-old patient were continuously tracked from waking to sleeping; (**c**) waking of the patient; (**d**) during sleep, the patient breathed more deeply (higher amplitude) and slowly (14 times per minute) than when awake (20 times per minute); (**e**) breathing patterns of this patient while sleeping with piano music; the person breathed more regularly and slowly (11 times per minute) than when sleeping without music (14 times per minute) (**d**). The abnormal breathing observed around 45 s almost disappeared when sleeping with music.

Breathing is vital to life. Therefore, the real-time monitoring of a patient’s breathing pattern is crucial to the support of respiratory rehabilitation therapies, such as magnetic resonance exams for respiratory-triggered imaging, chronic pulmonary disease treatment, and synchronized functional electrical stimulation. While several respiratory monitoring devices have already been developed [91,92,93,94,95,96], they are often in direct contact with a patient, which increases the chance of inaccurate or limited data. In this context, Thiabgoh et al. [94,96] developed a novel, noninvasive, and contactless magnetic sensing platform based on magneto-LC resonance (MLCR) technology that can precisely monitor a patient’s breathing, movement, or sleep patterns, thus providing efficient monitoring at a clinic or at home. A combination of the GMI and LC-resonance effects makes the MLCR sensor extremely sensitive to small variations in magnetic field. By placing a tiny permanent magnet on a patient’s chest, the MLCR sensor can precisely convert the magnetic oscillations generated by the patient’s breathing into an impedance spectrum (Figure 11a), which allows deep analysis of breathing variation to help identify respiratory-related diseases. Hwang et al. recently reported that the MLCR sensor can yield a distinct breathing pattern for each person tested and reveal abnormal breathing [95]. They showed that when individuals get older, they manifest weaker breathing marked by increased respiration rate. Older individuals held their breath for shorter periods of time and often experienced more respiratory issues than younger people. They also observed that as people became more relaxed (Figure 11b), their breathing occurred more regularly, and eventually more slowly while sleeping (Figure 11c) as compared to when awake (Figure 11d). Listening to relaxing music while sleeping was also found to help the patient breathe more regularly and slowly (Figure 11e) than in the case without music (Figure 11d) [95]. Research has shown that music can slow down our breathing rates, which helps to release stresses and promote relaxation as well as treat chronic pulmonary disease [97,98]. Research also has shown that breath-training techniques can help us relax, sleep, and breathe more naturally and effectively [99]. It is therefore anticipated that the ultrasensitive MLCR monitor can provide not only valuable information on a patient’s current health status, but also a novel breathing control tool for improving our health and physical performance.

**Figure 12:**
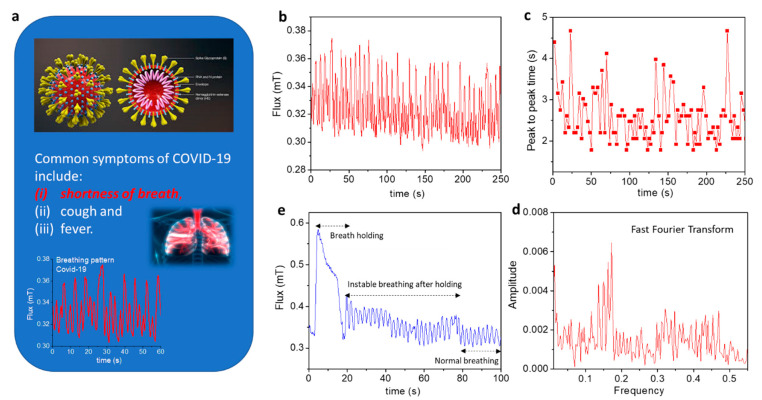
(**a**) Illustration of COVID-19 symptoms; top: schematic of the virus, bottom: breathing pattern of a patient with COVID-19. (**b**) Breathing pattern of the COVID-19 patient revealed irregular amplitude and several breathing anomalies. (**c**) Shortness of breath is evident from analysis of the peak-to-peak time versus measurement time. (**d**) The broad distribution of frequencies deduced from Fourier transform also reveals these features. (**e**) The COVID-19 patient lost the ability to hold his/her breath for a long time and required a much longer time to return to normal breathing.

COVID-19 has killed more than 80 million people around the world, a number which continues to increase daily. This outbreak represents an unprecedented global public health challenge. To limit the spread of COVID-19 and to help doctors in clinical decision making, detection and real-time monitoring of symptoms during early, intermediate, and severe states is critical. However, most of the existing detection methods yield limited information with long processing time, and often require significant amounts of sample data from the subject, which requires human contact [100,101,102]. Therefore, there is an urgent demand for developing contactless devices that enable early and fast detection of COVID-19 and to track its growth rate in real time. Recall that common symptoms of COVID-19 include (i) shortness of breath or difficulty breathing, (ii) cough, and (iii) fever (Figure 12a). Since the MLCR sensor can detect abnormal breathing, it can be employed to distinguish breathing patterns of healthy and COVID-19-infected individuals as well as to track in real time breathing pattern variations of COVID-19 patients at different stages of illness [103]. Phan’s group demonstrates the excellent capacity of using this technology to reveal shortness of breath and abnormal breathing in the breathing patterns of COVID-19 patients (Figure 12b–e) that are often absent in healthy people. It is worth mentioning that the tested COVID-19 patient lost the ability to hold his/her breath for extended periods and required a much longer time to return to a regular breathing pattern (Figure 12e) as compared to healthy people. Combining this technology with AI and machine learning, it could be possible to determine a COVID-19 patient’s health status (early, intermediate, or severe) and help propose an appropriate medical treatment plan based on the available data [100].

## 5. Concluding Remarks and Future Outlook

While magnetoimpedance-based biosensors show remarkable sensitivity at room temperature, much of the work performed in the detection of biomarkers remains qualitative. The ability to quantify magnetic and nonmagnetic biomarkers is key for the application of magnetoimpedance sensors beyond the detection of magnetic fields. In this respect, amorphous ribbons suffer from poor reproducibility due to the rapid-quenching fabrication process. We previously discussed the work by Devkota et al. that demonstrates the potential of introducing patterned holes into the surface of ribbons for improving their sensitivity. This approach warrants further study, and we propose that even the introduction of a single micron/nano-sized hole should have a significant impact on the detection sensitivity of ribbon-based magnetoimpedance biosensors. The interaction between magnetic particles and patterned holes of comparable sizes could permit detection of a single particle. While patterned holes can enhance the sensitivity of such sensors, reproducibility remains an issue that should be addressed by future studies. Multilayered films are the clear choice for quantifiable and reproducible sensors that can be mass produced due to their well-established fabrication techniques. Further work is necessary to enhance the detection limit of these sensors, which could be achieved by improving signal filtering to reduce noise, expanding fabrication techniques, and/or exploring different sensor geometries.

Beyond the detection of magnetic biomarkers, magnetoimpedance-based sensors show ultrahigh sensitivity to ultrasmall magnetic fields. Magnetic microwires show significantly higher sensitivity than ribbons or multilayered films but lack a platform for contact detection of magnetic biomarkers. However, their ultrahigh sensitivity to stray fields allows quantification of the stray field of magnetic nanoparticles, both static and in flow, as well as the detection of biomagnetic fields. This highlights the importance of choosing an appropriate magnetoimpedance-sensing element based on the desired application and is a testament to their versatility.

We have seen an increasing number of reports of GMI sensors for human healthcare monitoring. An interesting application that stands out is the work by Hwang et al. that uses a GMI-based magnetic field sensor to measure the respiratory motion of human subjects in a completely contactless manner. The authors further proposed using this technique to track the progress of COVID-19 patients and to determine if there is a correlation between different stages of COVID-19 and respiratory patterns. AI and machine learning have proven to be powerful tools to analyze these types of data, and in conjunction with other indicators such as heart rate, blood oxygen, etc., could be used for future diagnosis of COVID-19 patients. This model could be expanded to study several other respiratory illnesses, sleeping disorders, and healthcare tracking to offer a wide range of potential applications for novel GMI biosensors for human healthcare.

Given the fact that music can help us relax and improve our breathing, the development of magnetic–musical biofeedback for breathing regulation and control appears to be a novel approach that will stimulate further studies to fully exploit its unique practicality and wide-ranging healthcare monitoring applications. This technology can also find its place among other important applications in artificial intelligence, emotion detection, and space control and management.

The fast Fourier transform (FFT) algorithm, which converts a signal from the time domain to the frequency domain and vice versa, can be used in future research to extract spectral features from breathing patterns. This would also be used in combination with machine learning and other algorithms for signal processing to provide comprehensive information on a patient’s health status and physical performance.

## Figures and Tables

**Table 1:** Thin-film structures and MI ratios (%).

Structure (Thickness)	Dimensions	Max MI Ratio (%)	Max Sensitivity	MP Detection Applications	Ref.
		**Straight line**			
[Py(100 nm)/Ti(6 nm)]_4_/Cu(400 nm)/[Ti(6 nm)/Py(100 nm)]_4_	10 mm × 0.5 mm 1.5 mm × 90 microns	350 220	300%/Oe 75%/Oe	N/A	[68]
[Fe_21_Ni_79_(100 nm)/Cu(3 nm)]5/Cu(500 nm)/[Cu(3 nm)/Fe_21_Ni_79_(100 nm)]_5_	10 mm × 0.5 mm	160	41%/Oe	Stray field of MP in blood vessels	[28]
[Fe_19_Ni_81_(50 nm)/Ti(6 nm)]_6_/Cu(500 nm)/[Ti(6 nm)/Fe_19_Ni_81_(50 nm)]_6_	1 mm × 10 mm	~135	0.4 Ω/Oe	N/A	[69]
[FeNi(170 nm)/Ti(6 nm)]_3_/Cu(500 nm)/[Ti(6 nm)/FeNi(170 nm]_3_/Ti(6 nm )	10 mm × 0.5 mm		50%/Oe	Ferrogel detection	[70]
		**Meander**			
[Py(100 nm)/Ti(6 nm)]_4_/Cu(400 nm)/[Py(100 nm/Ti(6 nm)]_4_	5 mm × 4 mm, 12 strips 0.16 mm wide each	53.5	5.1 Ω/Oe	N/A	[71]
[FeNi(100 nm)/Cu(3 nm)]_4_/FeNi(100 nm)/Cu(500 nm)[FeNi(100 nm)/Cu(3 nm)]_4_/FeNi(100 nm)	200 microns wide, 14 strips 300 microns wide, 10 strips	60 165	-	Detection of polymer/MNP composites	[72]
Fe_17_Ni_83_(2 microns)/Cu(140 microns)/Fe_17_Ni_83_(2 microns)	5 mm long, 3 turns 6 turns	55.2 161.6	-	N/A	[73]
NiFe/Cu/NiFe	5 mm long 1.26 mm wide, 3 turns	97.54	0.1 μg mL^−1^ DPA concentration, 1 ng/mL AFP concentration	Dynabeads protein A, Alpha-fetoprotein detection	[61,74]

## Data Availability

Not applicable.
